# Development and Application of an In-Capillary CE-DAD Method for the Inhibitory Screening of Natural Extracts Towards Acetylcholinesterase Enzyme

**DOI:** 10.3390/metabo15040283

**Published:** 2025-04-18

**Authors:** Francesca Rinaldi, Sofia Salerno, Elena Frigoli, Giulia De Soricellis, Gloria Brusotti, Stefano Negri, Matteo Radice, Francesca Merlo, Andrea Speltini, Hellas Cena, Enrica Calleri

**Affiliations:** 1 Department of Drug Sciences, University of Pavia, Viale Taramelli 12, 27100 Pavia, Italy; francesca.rinaldi@unipv.it (F.R.); sofia.salerno01@universitadipavia.it (S.S.); elena.frigoli01@universitadipavia.it (E.F.); giulia.desoricellis@unipv.it (G.D.S.); gloria.brusotti@unipv.it (G.B.); 2 National Biodiversity Future Center (NBFC), 90133 Palermo, Italy; stefano.negri@univr.it (S.N.); hcena@unipv.it (H.C.); 3 Department of Biotechnology, University of Verona, 37134 Verona, Italy; 4 Campus Puyo, Universidad Estatal Amazónica, Km 2, Via Puyo-Tena, Puyo 160150, Ecuador; mradice@uea.edu.ec; 5 Department of Chemistry, University of Pavia, Via Taramelli 12, 27100 Pavia, Italy; francesca.merlo@unipv.it (F.M.); andrea.speltini@unipv.it (A.S.); 6 Clinical Nutrition and Dietetics Service, Unit of Internal Medicine and Endocrinology, ICS Maugeri IRCCS, 27100 Pavia, Italy; 7 Laboratory of Dietetics and Clinical Nutrition, Department of Public Health, Experimental and Forensic Medicine, University of Pavia, Via Bassi 21, 27100 Pavia, Italy

**Keywords:** Alzheimer’s disease, plant extracts, capillary electrophoresis

## Abstract

**Background:** The enzymatic activity of acetylcholinesterase (AChE) has been a focal point in neurodegenerative diseases research, particularly in relation to Alzheimer’s disease. This is attributed to the significantly reduced levels of cholinergic neurons observed in Alzheimer’s patients compared to healthy individuals. The strategy to mitigate the onset of these diseases in patients lies in the exploration of new potential AChE inhibitors with a focus also on natural extracts. A rapid and specific capillary electrophoresis method with direct ultraviolet detection (CZE-UV/Vis) was developed to screen natural extracts by assessing their potential to inhibit AChE. **Materials and Methods:** To enhance the specificity when analysing complex matrixes such as natural extracts, a sequential analysis approach based on the “sandwich model” was implemented using Ellman’s reagent [5,5′-dithiobis-(2-nitrobenzoic acid)] (DTNB) as a colorimetric indicator. **Results:** A reference inhibitor, neostigmine, was used for system validation through IC_50_ and K_i_ values determination by subsequent injections of acetylthiocholine substrate in the presence of neostigmine at increasing concentrations, and the enzyme combined with DTNB in borate-phosphate buffer (30 mM, pH 8.0). The enzymatic product was selectively detected at 412 nm. The validated system was applied to the analysis of seven natural extracts. **Conclusions:** Results demonstrated promising outcomes for identifying phytotherapeutic agents with potential applications in the prevention of neurodegenerative diseases. This method provides high selectivity and automation, offering a streamlined and effective approach for screening natural matrices containing potential AChE inhibitors.

## 1. Introduction

The study of the role of enzymes in pathological states and their pathways is a crucial aspect in the process of the discovery of new drugs. Among them, acetylcholinesterase provides a promising approach into the treatment of neurodegenerative diseases, particularly Alzheimer’s disease [1,2]. AChE, a key enzyme of the cholinergic system located at the level of the post-synaptic membrane, catalyses the hydrolysis of acetylcholine into acetic acid and choline, which is reabsorbed by the post-synaptic neuron under physiological conditions, thus terminating nerve transmission and regulating ACh levels at the synaptic cleft [3]. In Alzheimer’s disease, characterised by a progressive loss of cholinergic neurons [4], the already diminished levels of ACh due to neuronal degeneration are further reduced. To counteract this process, inhibitors of the enzyme acetylcholinesterase have been identified [1]. The mechanism of action of AChEI involves binding to the enzyme reducing its hydrolytic activity. Consequently, ACh remains active in the synaptic cleft for a longer time, leading to increased levels, partial restoration of cholinergic function and enhanced neuronal communication [3,5]. The study of enzyme inhibitors is often carried out by methods such as absorption or fluorescence spectroscopy, which can be easily automated using commercial 96-well plates. However, these techniques are only applicable when substrates and enzyme products have significant differences in their spectrophotometric properties, a rare feature in this type of reaction. Furthermore, the fluorescent signal can be compromised by background absorption and self-fluorescence of the biological samples [5]. These problems could be solved by liquid chromatography (LC) coupled with mass spectrometry (MS), which allows the separation of the reaction mixture into its individual components. This technique is particularly suitable for enzyme assays, facilitating both the evaluation of enzymatic activity and inhibition by small molecules. Nevertheless, this approach presents certain limitations, including the complexity of the separation optimisation, long elution times and high costs due to the high percentage of waste [5]. Furthermore, a reversed phase LC is often exploited, which does not allow to replicate the physiological conditions in which the enzyme would work [6].

Capillary electrophoresis is a valid alternative to overcome all these limits as it offers optimal results and efficiency, all due to its short analysis time, low reagents cost and minimum sampling requirement. In addition, CE could be combined with various detection techniques including UV, laser induced fluorescence (LIF), electrochemical detectors and MS [7]. As a miniaturised separation technique, CE offers numerous advantages. Among these, UV-detection capillary zone electrophoresis (CZE-UV) is known to be fast, precise and easy to perform. The techniques for screening enzyme inhibitors with CE can generally be distinguished into pre-capillary enzyme analysis and in-capillary enzyme analysis. In pre-capillary enzyme assays, the enzymatic reaction is carried out by mixing the enzyme, substrate and any necessary cofactors outside the CE system. The reaction occurs offline, and the solution is incubated for a specific period [8]. Upon completion, the reaction is halted by adding quenching reagents or changing the conditions of the reaction [9]. After this, the products are separated and detected by the CE system following the injection of the mixture [8]. This method is relatively simple, and the independent execution of the reaction and analysis allows each step to be optimised without interference [8].

In the in-capillary enzyme assay, the capillary acts as both separation channel and microreactor [10]. This configuration allows reagent injection, mixing, enzyme reaction, separation and detection within a single capillary, thus increasing the efficiency of the analysis [8], automating the process and reducing analysis time and costs. In-capillary enzyme assay can be classified into two main types: electrophoretic mediated microanalysis (EMMA) and immobilised enzymatic micro reactor (IMER) [5,11]. EMMA involves mixing enzymes and substrates directly into the capillary and operates in two different modes. The first of these, known as continuous engagement or long contact mode, involves filling the capillary with the enzyme or substrate while the second reagent is introduced as a plug using zonal sample introduction or in-continuous flow through moving boundary sample introduction [11]. When a voltage is applied, electrophoretic mixing and the enzymatic reaction occurs. Alternatively, in the EMMA transient engagement (plug-plug or short contact mode) the enzyme and substrate are introduced sequentially into the capillary and the reaction is jump-started by the applied voltage, as the reagents mix according to their electrophoretic mobility [5]. This second mode is considered more suitable compared to the previous one because it consumes fewer reagents. Nonetheless, it is essential that the buffer is compatible with both the enzymatic reaction and separation process. However, to overcome this issue, one approach is to add a reaction buffer or water injections that create a separation between the reaction space from the run-through buffer. Alternatively, the IMER technique relies on the immobilisation of enzymes in the initial section of the capillary for the reaction, while the rest is responsible for the separation of analytes [7] or in all the capillary only if the background electrolyte (BGE), a buffer solution, has the same conditions as the enzymatic reaction [5]. Immobilisation allows enzymes to be reused and minimises the possibility of enzymatic adsorption on the capillary wall. However, IMER is not suitable when the incubation and separation requires different buffers [7].

In the context of research into AChEI, natural extracts have emerged as a promising and valuable resource for the identification of small molecules [10,12]. For this reason, various CE methods have been reported in the literature for assessing the inhibitory effects of plant-derived extracts on AChE. One approach, proposed by Siebert et al. [13], involves the immobilisation of AChE onto MnFe_2_O_4_ magnetic nanoparticles using chitosan and glutaraldehyde as cross-linking agents. This strategy allows for high enzyme-loading capacity and facilitates easy insertion and removal of the enzyme from the capillary. However, despite these advantages, the technique suffers from low reproducibility [14]. Min et al. [15] introduced another method that utilises a microreactor positioned at the injection end of the capillary. Here, AChE is immobilised onto the inner surface of a fused-silica capillary previously coated with polyethylenimine. The negatively charged enzyme interacts electrostatically with the positively charged capillary coating, while chitosan is added to enhance binding stability. However, since this immobilisation relies on relatively weak physical interactions, it may lead to progressive enzyme loss over time. Tang et al. [16] proposed a method for the screening of acetylcholinesterase inhibitors in natural extracts by CE. However, the screening was carried out at low extract concentration (0.5 mg/mL) and false negative results cannot be excluded. The same authors proposed a capillary electrophoresis-based approach in which AChE is immobilised on the capillary walls using a layer-by-layer deposition technique. This method results in an immobilised capillary enzyme reactor, offering enzyme stability and reusability. However, a limitation of this technique is the relatively low enzyme-loading capacity [17]. Zhao et al. [10] explored the use of a solid support material as a carrier for AChE immobilisation, selecting chitosan-modified cellulose filters as the substrate. The presence of amine groups on the modified paper enables enzyme attachment. This material is biocompatible, has a high specific surface area, is cost-effective and environmentally friendly. Nevertheless, the immobilisation process is complex and time-consuming. To address the challenges associated with enzyme immobilisation, Campos et al. [18] proposed an alternative CE-based approach for screening AChE inhibitors. This method utilises EMMA in an online in-capillary format. By applying a hydrodynamic “sandwich” injection, the inhibitory effect of plant extracts can be evaluated by tracking changes in the peak area of the reaction product, thiocholine, at 230 nm. While effective, this approach lacks specificity, as signals from the reaction product can overlap with those from the unreacted substrate and plant extract components.

To improve specificity, with the idea to develop a reliable and fast method for the screening of natural extracts, we developed and validated a new and fast in-capillary method using Ellman’s reagent [19]. Ellman’s reagent enables enzyme activity measurement through a colorimetric reaction, producing a detectable signal at 412 nm. The system was first validated with a known inhibitor, neostigmine [18]. The method was then applied to real samples, methanolic extracts of plants with known and unknown inhibitory activities.

The method has proven to be robust and easy to apply and can be successfully used for the rapid screening of large numbers of extracts.

## 2. Materials and Methods

### 2.1. Reagents and Chemicals

Acetylthiocholine (AThCh), acetylcholinesterase (AChE), 2,2′-dinitro-5,5′-dithiobenzoic acid (DTNB), neostigmine, boric acid (H_3_BO_3_), dichloromethane (DCM), sodium dodecyl sulfate (SDS) and magnesium sulfate (MgSO_4_) were purchased from Sigma-Aldrich (St. Louis, MO, USA). Neostigmine was purchased from Merck KGaA (Darmstadt, Germany).

The solvents methanol (MeOH) and acetonitrile (ACN) were obtained from PanReac AppliChem ITW Reagents (Cinisello Balsamo, Italy). Sodium phosphate monobasic (NaH_2_PO_4_) and acetone were purchased from Carlo Erba Reagents (Cornaredo, Italy).

Deionised water used in the experiments was obtained using the Milli-Q^®^ Integral purification system by Merck KGaA (Darmstadt, Germany).

A 1 M sodium hydroxide (NaOH) solution was supplied by Agilent Technologies (Santa Clara, CA, USA).

### 2.2. Sample Preparation

#### 2.2.1. Stock and Working Solutions

A stock solution of the substrate AThCh was prepared at a concentration of 50 mM in 30 mM borate–phosphate buffer at pH 8.0. This stock solution was subsequently properly diluted in buffer to prepare working solutions.

A stock solution of the enzyme AChE was prepared at a concentration of 100 U/mL in 30 mM borate–phosphate buffer at pH 8.0. A stock solution of DTNB was also prepared at a concentration of 6.67 mM in the same buffer. Both stock solutions were appropriately diluted to prepare a working solution containing AChE at a concentration of 5 U/mL and DTNB at a concentration of 3.33 mM.

#### 2.2.2. Plant Extracts Preparation

The plant extracts were kindly provided by Flavia Guzzo, University of Verona, and are part of the Phytocomplexes of the Mediterranean collection of the National Biodiversity Future Center. The leaves were collected from one individual of each of the following species grown in the Botanical Garden of Padova (Italy): *Aloysia citrodora* Paláu (Verbenaceae), *Artemisia abrotanum* L. (Asteraceae), *Castanea sativa* Mill. (Fagaceae), *Diospyros kaki* Thunb. (Ebenaceae) and *Rosa canina* L. (Rosaceae). For *Butomus umbellatus* L. (Butomaceae), also growing in the Botanical Garden of Padova, leaves and stems were collected from five individuals, whereas *Beta vulgaris* L. (Amaranthaceae) was grown in the greenhouse facility of the University of Verona and leaves were collected from six individuals. Samplings were performed in triplicate. Upon collection, the plant material was immediately frozen in dry ice. It was then ground with liquid nitrogen using an A11 basic analytical mill (IKA-Werke, Staufen, Germany) and 1 g of the resulting frozen powder was extracted with 10 mL of LC-MS grade methanol (Honeywell, Seelze, Germany). The samples were vortexed for 30 s, sonicated on ice for 10 min in a 40-kHz ultrasonic bath (SOLTEC, Milano, Italy) and centrifuged at 14,000× *g* for 10 min at 4 °C. Supernatant was split into 1 mL-aliquots, each corresponding to 100 mg of fresh material, and dried with a speed-vac system (Heto-Holten; Frederiksborg, Denmark).

The dry extract was first diluted in 1 mL of DCM and dried under nitrogen to obtain the accurate weight. After determining their actual weight, a stock solution was prepared at a concentration of 20 mg/mL in methanol (MeOH). This stock solution was centrifuged for 5 min at 13,000 rpm and 25 °C using the Centrifuge 5804 R Eppendorf (Amburgo, Germania). The supernatant was collected to prepare a working solution with a concentration of 10 mg/mL and added with 10 mM AThCh. The negative control solution was prepared by replacing the plant extract with an equal volume of methanol.

### 2.3. Electrophoretic System and Methods

#### 2.3.1. Instrumentation and Operating Conditions

The instrument used for the analyses was the Agilent 7100 Capillary Electrophoresis System (Santa Clara, CA, USA), equipped with a polyimide-coated fused silica capillary with an internal diameter of 50 μm, an external diameter of 363 μm, a total length of 48.5 cm and an effective length of 40 cm. Electrophoretic separation was performed using a BGE composed of 30 mM borate–phosphate buffer, pH 8.0. To condition a new capillary, the following sequence was used: 1 M sodium hydroxide for 10 min, 0.1 M sodium hydroxide for 10 min, water for 10 min and 30 mM borate–phosphate buffer at pH 8.0 for 15 min. Daily conditioning of the capillary was carried out with 30 mM borate–phosphate buffer at pH 8.0. Analyses were carried out at a temperature of 37 °C, applying a voltage of 25 kV to enable electrophoretic separation of the reaction products. The analysis time was 15 min, preceded by 5 min of buffer conditioning and followed by washing with acetonitrile to remove any residual enzyme, extract or inhibitor. Detection was performed at a wavelength of 230 nm, corresponding to the absorption maximum of thiocholine, and at 412 nm, specific for the absorption of the 2-nitro-5-thiobenzoic acid anion. The wash between the analyses was carried out with running water for 3 min, acetonitrile for 3 min, water for 3 min and finally buffer for 3 min with the flush mode.

#### 2.3.2. Injection Method

**Figure 1:**
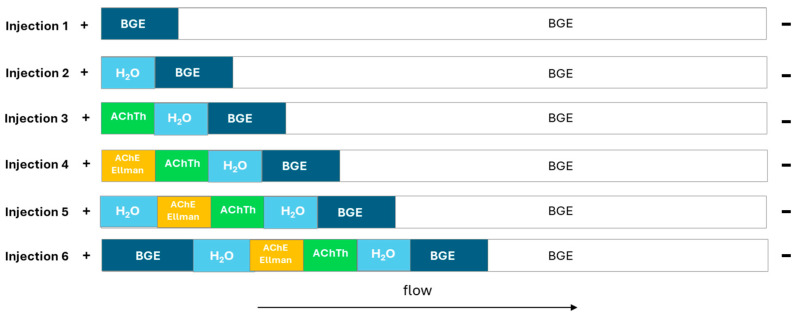
Description of plugs in the sandwich model for enzymatic reactions in the evaluation of enzyme activity.

A hydrodynamic “sandwich” injection mode was employed, which involves, in sequence, the injection of BGE (50 mBar, 10 s), water (20 mBar, 5 s), acetylcholinesterase enzyme and DTNB solution (50 mbar, 5 s), followed by the acetylthiocholine substrate solution (eventually with neostigmine as the positive control or natural extracts for the inhibition studies) (50 mBar, 5 s), water (20 mBar, 5 s) and finally BGE (50 mBar, 10 s). The injection end of the capillary is rinsed with water between the enzyme and substrate injections to avoid contamination (0 mBar, 5 s). The choice to begin and end the injection with water is necessary to create a reaction environment suitable for the enzyme activity. The injection diagram in reported in Figure 1.

After the injections, a waiting time of 2 min is applied, allowing the reagents to interact and reactions to occur. Subsequently, the application of the potential difference allows the separation of the reaction products. To avoid any possibility of contamination between enzyme and substrate samples, a washing step of the capillary inlet simulating an injection of water (0 mbar, 5 s) was included.

### 2.4. Kinetic Study

Working solutions of the substrate AThCh were prepared by diluting the 50 mM stock solution in 30 mM borate–phosphate buffer, pH 8.0 at concentrations of 0.625 mM, 1.25 mM, 2.5 mM, 5 mM and 10 mM. The concentrations of Ellman’s reagent and AChE enzyme were kept constant at 3.33 mM and 5 U/mL, respectively.

The Michaelis–Menten constant (*K_m_*), expressed in mM, was determined using the Prism software version 10.4.1 (GraphPad, San Diego, CA, USA) through nonlinear regression and applying the Michaelis–Menten enzyme kinetics equation:

(1) $$Y=\frac{V_{max}\;\times \;X}{K_{m}+X}$$

where *V_max_* is the maximum reaction rate of the enzyme.

The *K_m_* value was calculated based on the substrate concentration *X* (in mM) and the reaction rate *Y*, determined as the ratio between the peak area of the reaction product (the anion of TNB) at 412 nm and the reaction time of 2 min, as reported by Calleri et al. [19]:

(2) $$Reaction\;Rate=\frac{Area\;(mAU\;\times \;s)}{Reaction\;Time\;(min)}$$

### 2.5. Inhibition Study

The inhibition curve was generated by the analysis of working solutions consisting of AThCh 10 mM, prepared by diluting the 50 mM AThCh stock solution in buffer, and the inhibitor neostigmine at increasing concentrations of 0.4 μM, 4 μM, 8 μM, 20 μM, 40 μM, and 200 μM, prepared by dilution of a 10 mM neostigmine stock solution in 30 mM borate–phosphate buffer, pH 8.0. The concentrations of Ellman’s reagent and AChE enzyme were kept constant at 3.33 mM and 5 U/mL, respectively. The percentage of inhibition was calculated by comparing the reaction performed with and without neostigmine, as reported by Bartolini et al. [20]. The chromatograms were read at the wavelength of 412 nm, and the percentage of inhibition was calculated by the ratio between the areas of the TNB anion in presence and in absence of the inhibitor; the ratio is then subtracted from 100 to obtain the inhibition percentage:

(3) $$\%\;Inhibition=100-\left(\frac{A_{i}}{A_{0}}\right)\times 100$$

where *A_i_* is the area in presence of neostigmine and *A*_0_ is the area in absence of neostigmine.

The inhibition constant (*Ki*), expressed in M, was determined using the Prism version 10.4.1 software (GraphPad, San Diego, CA, USA), employing the “One Site–Fit Ki” model and applying the equations:

*logIC*_50_ = *log*(10^logKi*(1 + [*AThCh*]/*AThCh K_m_*)) (4)

*Y* = *Bottom* + (*Top* − *Bottom*)/(1 + 10^(*X* − *LogIC*_50_)) (5)

IC_50_ is the value of the inhibitor concentration (neostigmine), expressed in M, required to achieve 50% inhibition. [AThCh] is the constant substrate concentration (expressed in nM). AThCh *K_m_* is the Michaelis–Menten constant of the substrate (expressed in nM). Top and Bottom represent the plateaus reached on the *Y*-axis.

The *Ki* value is calculated with the logarithm of the neostigmine inhibitor concentration (*X*), expressed in M, and the percentage of residual activity (*Y*), determined as the percentage ratio of the area of the reaction product (TNB anion) at a wavelength of 412 nm in reactions conducted with and without the inhibitor:

(6) $$\%\;Residual\;Activity=\frac{A_{i}}{A_{0}}*100$$

$$A_{i}=Area\;in\;presence\;of\;neostigmine,\;A_{0}=Area\;in\;absence\;of\;neotigmine$$

The IC_50_ value was determined using the “One Site–Fit IC_50_” model in Prism version 10.4.1 software (GraphPad, San Diego, CA, USA), using the following equation:

*Y* = *Bottom* + (*Top* − *Bottom*)/(1 + 10^(*X* − *LogIC*_50_)) (7)

### 2.6. Screening of Plant Extracts

To evaluate the inhibitory power of the pull of natural extracts, analyses of the reaction between the AThCh substrate (10 mM), AChE enzyme (5 U/mL) and DTNB (3.33 mM) conducted in the presence and absence of each extract were compared. After each analysis, a washing step with acetonitrile was performed. The inhibition percentage was determined as the percentage ratio between the area of the peak of the TNB reaction product, detected at a wavelength of 412 nm, in the reactions conducted with and without the extract subtracted from 100. For each plant, three replicates were performed, calculating the average value and standard deviation.

(8) $$\%\;Inhibition=100-\left(\frac{A_{i}}{A_{0}}\right)*100$$

$$A_{i}=Area\;in\;presence\;of\;extract,\;A_{0}=Area\;in\;absence\;of\;extract$$

## 3. Results

**Figure 2:**
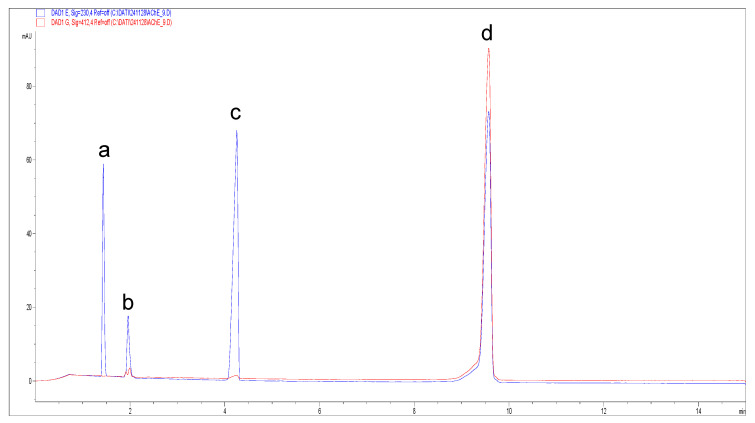
Electropherograms from the AChE reaction detected at 230 nm (blue trace) and 412 nm (red trace). The reaction was carried out using 10 mM AThCh and 5 U/mL AChE in presence of 3.33 mM Ellman’s reagent (DTNB). Background electrolyte (BGE): 30 mM borate-phosphate (pH 8.0). Capillary electrophoresis (CE) operational conditions: voltage 30 kV; wavelengths 230 nm and 412 nm; temperature 37 °C. Ordered multiple injection: 50 mBar at 10 s (BGE); 20 mBar at 5 s (water); 50 mBar at 5 s (AChE 5 U/mL); 0 mBar at 5 s (water); 50 mBar at 5 s (AThCh); 20 mBar at 5 s (water); 50 mBar at 10 s (BGE). Capillary dimension: I.D. 50 μm, 40.0 cm of effective length, and 48.5 cm total length. Peak assignment: peak (**a**) substrate AChTh, peak (**b**) adduct formed from the reaction between TNB and Ach, peak (**c**) unreacted Ellman’s reagent, peak (**d**) TNB.

The use of AThCh as substrate and Ellman’s reagent in the hydrodynamic “sandwich” injection mode (see Section 2.3.2), allows to obtain the eletropherogram reported in Figure 2 detected at 230 nm (blue trace) and 412 nm (red trace).

### 3.1. Kinetic Studies

Kinetic parameters, describing the activity of the AChE enzyme were first derived following Section 2.4. The injection was carried out following the “sandwich” hydrodynamic injection scheme described in Section 2.3.2.

The reaction was carried out at increasing substate concentrations, and three replicates were made for each concentration level. Table 1 reports the average areas of TNB obtained at each AThCh concentration level.

**Figure 3:**
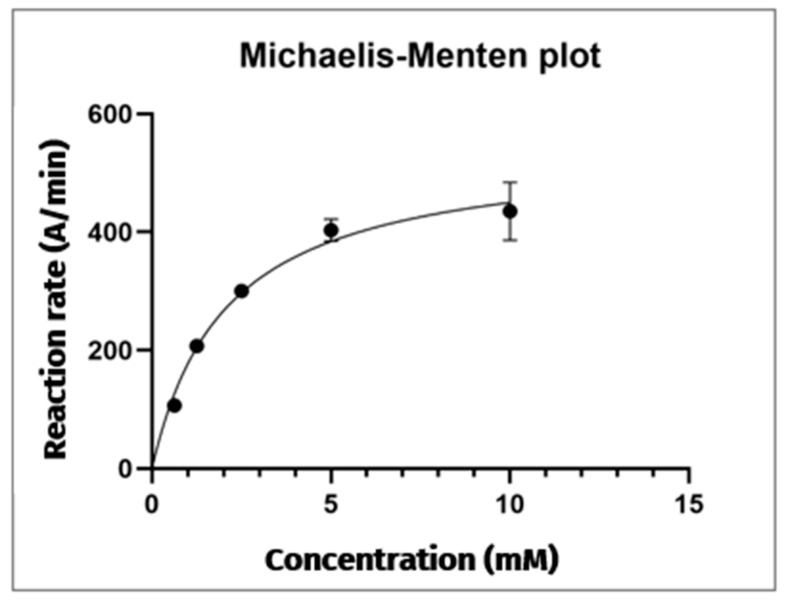
Michaelis–Menten plot of the AChE reaction. Each point is the mean of three replicates, with error bars indicating ± standard deviation.

Comparison of the electropherograms from each analysis reveals that the area under the reaction product peak increases proportionally with the substrate concentration used. The Michaelis–Menten plot was constructed with the substrate concentration on the x-axis and the reaction rate (defined as the average area of the TNB anion peak at λ = 412 nm/reaction time) on the y-axis (Figure 3). This graph displays a positively increasing trend, with a plateau reached at a substrate concentration of 10 mM. This observation confirms the saturation of the hydrolysis reaction catalysed by AChE (5 U/mL) on the AThCh substrate. Beyond this concentration, further increases in substrate availability do not affect the amount of TNB product formed. Hence, to carry out the subsequent inhibition studies, the hydrolysis reaction is carried out under the following conditions: AChE enzyme 5 U/mL, AThCh substrate 10 mM and DTNB 3.33 mM.

The curve is described by the following equation:

$$Y=\frac{543.1\;\times \;X}{2.07+X}$$

The data collected allowed the determination of kinetic parameters the Michaelis–Menten constant (*K_m_*) and the maximum reaction rate (*V_max_*), respectively.

For the AChE enzyme at a concentration of 5 U/mL, the obtained value of the *K_m_* is 2.07 mM, while the *V_max_* is 543.1 A/min.

### 3.2. Inhibition Studies

Neostigmine was used as a reference inhibitor to perform AChE inhibition studies [18]. Following the results obtained in Section 3.1, these studies were carried out in saturation conditions keeping the AChE concentration fixed at 5 U/mL and AThCh at 10 mM while increasing neostigmine concentration. Each reaction was performed by the hydrodynamic injection method described in Section 2.3.2. The area below the peak of the reaction product, anion TNB, at the wavelength of 412 nm, was determined for each neostigmine concentration levels. The results are shown in Table 2.

As expected, the increase in inhibitor concentration leads to a reduction in the area below the peak corresponding to the reaction product.

**Figure 4:**
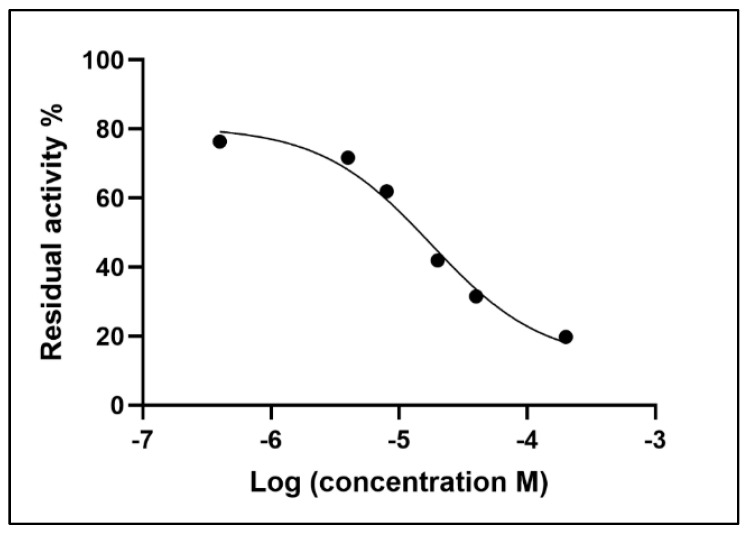
Inhibition curve for neostigmine towards AChE.

As shown in Figure 4, by plotting the logarithm of the inhibitor concentration on the *x*-axis and the percentage residual activity on the *y*-axis, a curve is obtained which describes the inhibitory behaviour of neostigmine vs. the AChE enzyme. As described in Section 2.5, the value of the inhibition constant (*Ki*) for neostigmine is 3.047 μM, while the inhibitory concentration (IC50) is 17.77 μM.

### 3.3. Evaluation of Inhibitory Activity of Natural Extracts

To evaluate the suitability of the method in the assessment of the inhibitory power of natural extracts from plant leaves, it was applied to the analysis of edible plant extracts with known AChE inhibitory activity. In this perspective, extracts from *A. citriodora* (lemon verbena), *A. abrotanum* (southernwood), *B. umbellatus* L., (flowering rush), *B. vulgaris* (beetroot), *C. sativa* (chestnut), *D. kaki* (persimmon) and *R. canina* (dog rose) have been analysed.

**Figure 5:**
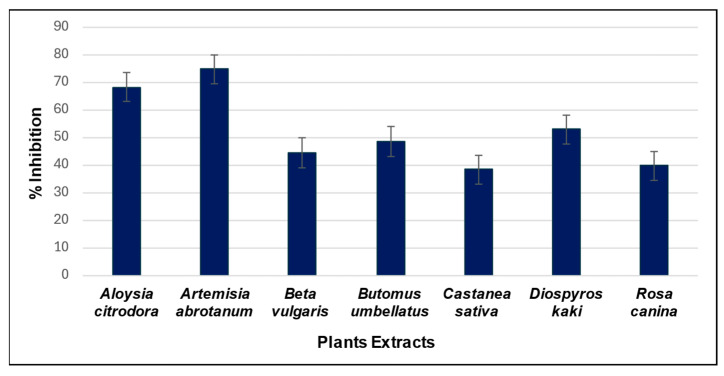
Histogram showing the inhibitory power of the analysed extracts. Each column represents the % inhibition of the respective extract, calculated on analyses performed in triplicate.

**Figure 6:**
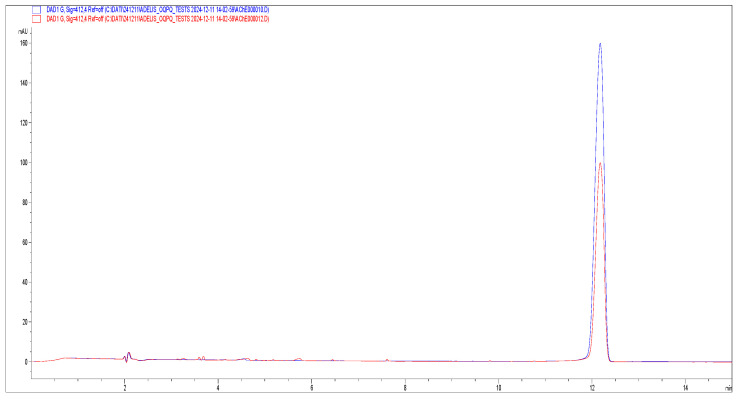
Electropherograms of TNB peak detected at λ = 412 nm, obtained from the analysis of the reaction between substrate AThCh 10 mM, enzyme AChE 5 U/mL, Ellman reagent 3.33 mM in presence of methanolic extract of *B. umbellatus* leaves 10 mg/mL (red) and in the presence of an equal volume of methanol (blue).

The inhibitory potential of this pool of natural extracts against AChE was assessed by incorporating each extract into samples containing AThCh, following the “sandwich” injection method described in Section 2.3.2. To account for the presence of methanol in the sample containing both the substrate AThCh and the extract, the control sample (lacking the extract) was prepared by adding an equivalent amount of methanol. This approach ensures that any signals observed in the electropherogram due to methanol are consistent across samples, thereby allowing for accurate comparison between the electropherograms. For each plant, three replicate analyses were performed in the presence and absence of the extract. Figure 5 shows the mean inhibitory percentage of each extract while Figure 6 reports, as an example, the electropherograms for the analysis of the reaction with and without the natural extract (*B. umbellatus* leaves).

The most promising results were obtained from reactions carried out in the presence of leaf extracts of *A. citriodora* and *A. abrotanum*, which showed a stronger inhibitory effect than the other extracts. Interestingly, we reported the inhibitory activity of the *B. umbellatus* extract for the first time. Finally, these three extracts showed promising potential for future explorations in AChE inhibition.

## 4. Discussion

The aim of this work was the development of a reliable and simple CE method for the rapid screening of AChE inhibitors in natural extracts. The assay was designed to provide a rapid and simple method, with minimum reagents and solvents consumption.

The reaction of the substrate acetylthiocholine with AChE to give thiocholine and acetic acid was evaluated as a reference assay. In order to use active extract concentrations and to avoid false negative results, Ellman’s reagent was introduced to increase the selectivity of the assay. A sandwich injection model was first selected [18] which involved the consecutive hydrodynamic injection of BGE, water, enzyme, substrate (also combined with inhibitors), again enzyme, water and BGE. Moreover, a waiting time of 2 min was introduced at the end of the injections to allow the reaction to take place, before applying a current of 30 kV and starting the electrophoretic migration. In these conditions, the observed peaks were split, as the result of two reaction windows. It was, therefore, decided to remove the second injection of enzyme to avoid peak splitting. The final sandwich included successive injections of BGE, water, enzyme, substrate (also combined with inhibitors), water and BGE (Figure 1). To avoid any possibility of contamination between enzyme and substrate samples, a washing step of the capillary inlet simulating an injection of water (0 mbar, 5 s) was included.

For the washing steps, we used an organic solvent-based procedure to break any non-specific interactions between the enzyme and the capillary walls. Acetonitrile was chosen because it effectively denatures the enzyme, making it easier to remove from the capillary. Analyses were carried out at two different wavelengths: 230 nm to detect all the species deriving from the AChE reaction and 412 nm to visualise only the peak of the yellow anion deriving from the reaction with DTNB.

A standard enzymatic assay to estimate kinetic parameters describing the activity of the AChE enzyme was first set-up by optimising enzyme concentration. Initially, enzyme kinetics was studied by fixing the concentration of AChE at 10 U/mL and using increasing concentrations of the AThCh substrate. However, under these conditions, too high concentrations of AChTh substrate were required, thus moving away from one of the advantages of the instrument, namely the possibility of using small amounts of reagents. The AchE concentration was therefore reduced to 5 U/mL.

After experimental parameters optimisation, the method was validated using a known inhibitor. Inhibition analysis was carried out using neostigmine as inhibitor standard compound. The IC_50_ value was found to be in agreement with the same values reported in the literature [13,21].

To evaluate the suitability of the method in the assessment of the inhibitory power of natural extracts, seven edible plant extracts, some of them with known AChE inhibitory activity, were selected.

As can be seen in Figure 5, all the analysed extracts showed an inhibitory effect against AChE. The most promising results were obtained from reactions performed in the presence of *A. citriodora* and *A. abrotanum*, which exhibited a stronger inhibitory effect compared to the other extracts.

A comparison of the proposed method with already known methods shows that the generally most widely used method is the Ellman’s method. This methodology is reported as the prevalent method in the review articles. By integrating Ellman’s reagent to the present validated method, specificity was enhanced while maintaining efficiency, making it a more robust and reliable tool for screening AChE inhibitors in natural extracts and avoiding more laborious methods based on cellular models.

Concerning the present study, *A. citriodora* and *A. abrotanum* leaf extracts have been reported the stronger inhibitory effect. According to the literature, all these extracts contain a variety of bioactive compounds, including phenols [22], kaempferol, different quercetin isoforms [23] and triterpenoids [17], which are well known for their ability to inhibit AChE. In the case of *A. citriodora,* some previous studies have already reported preliminary data on neuroprotective activity, anxiety and insomnia treatment [24,25,26].

Similarly, the effectiveness of *A. abrotanum* extract can be linked to its rich content of monoterpenoids, such as 1,8-cineole, artemisia ketone, cis-thujone, trans-thujone, cis-epoxycimene, anphor, borneol and cis-sabinyl acetate [27]. In addition, the genus Artemisia is characterised by the presence of the sesquiterpene lactone artemisinin, the flavonol Kaempferol and the flavonoid quercetin. All the above-mentioned compounds have shown promising AChE inhibitory activity and have been mentioned as potential therapeutic agent in Alzheimer’s disease [28,29,30,31]. After proving the suitability of the method in screening the AChE inhibitory activity of natural extracts, it was applied to a methanolic extract of *B. umbellatus* (flowering rush), for which no studies directly addressing the AchE inhibitory activity are available.

## 5. Conclusions

This study presents the development of a rapid and efficient CE method for assessing AChE inhibition, demonstrating both sensitivity and reliability in enzyme activity evaluation. By direct UV/Vis detection and sample injection through a “sandwich model” approach, the method effectively minimises reagent consumption while maintaining high analytical performance. The Ellman reagent, due to its high specificity for thiol groups, enables a sensitive and selective assessment of acetylcholinesterase inhibition, ensuring accurate kinetic measurements in our study.

Method suitability in the measurement of AChE inhibition was confirmed through the analysis of the reference inhibitor neostigmine, ensuring reliable IC_50_ and K_i_ determinations. When applied to natural extracts, the technique successfully identified several promising extracts with significant inhibitory potential. Among them, *A. abrotanum* exhibited the most notable activity, suggesting its potential as a source of bioactive compounds relevant to neurodegenerative disease treatment. Additionally, *A. citrodora* and *A. abrotanum* showed promising inhibition, reinforcing the importance of natural products in enzyme inhibition research. The inhibitory activity of *B*. *umbellatus* was demonstrated for the first time.

Overall, this study underlines the potential of CE-UV/Vis as an effective tool for screening AChE inhibitors, offering a reliable platform for natural product-based drug discovery. Moreover, this technique could be used to support a bioguided fractionation and isolation of the compounds responsible for the inhibitor activity.

The findings pave the way for further pharmacological studies, contributing to the ongoing search for novel neuroprotective agents.

## Figures and Tables

**Table 1:** Average values of the area under the TNB anion peak and the corresponding standard deviation (SD) for three replicates at each analysed concentration, λ = 412 nm.

[AThCh] (mM)	Area TNB (mAUxs)	SD Area TNB 412 nm (mAUxs)
0.625	213.800	6.855
1.25	414.733	6.014
2.5	600.367	7.146
5	806.967	37.100
10	870.767	98.146

**Table 2:** Values of the area below the TNB peak at 412 nm, inhibition percentage and residual activity percentage for each neostigmine concentration analysed.

[Neostigmine] (μM)	Area TNB (mAU*s)	Inhibition (%)	Residual Activity (%)
0.4	609.3	23.592	76.408
4	571.5	28.333	71.667
8	493.9	38.064	61.936
20	334.4	58.065	41.935
40	251.8	68.424	31.576
200	157.5	80.249	19.751

## Data Availability

The data presented in this study are available upon request from the corresponding author.

## Abbreviations

The following abbreviations are used in this manuscript:

AChE	Acetylcholinesterase
CZE-UV/Vis	Electrophoresis method with direct ultraviolet detection
DTNB	[5,5′-dithiobis-(2-nitrobenzoic acid)]
ACh	Acetylcholine
AChEI	Inhibitors of the enzyme acetylcholinesterase
CE	Capillary electrophoresis
CZE-UV	UV-detection capillary zone electrophoresis
EMMA	Electrophoretic mediated microanalysis
BGE	Background electrolyte
IMER	Immobilised enzymatic micro reactor
LbL	Layer-by-layer
AThCh	Acetylthiocholine
DMC	Dichloromethane
ACN	Acetonitrile
TNB	2-nitro-5-thiobenzoic acid
